# Dynamic navicular motion measured using a stretch sensor is different between walking and running, and between over-ground and treadmill conditions

**DOI:** 10.1186/s13047-015-0063-z

**Published:** 2015-02-25

**Authors:** Christian J Barton, Simon L Kappel, Peter Ahrendt, Ole Simonsen, Michael S Rathleff

**Affiliations:** Complete Sports Care, Melbourne, Australia; Lower Extremity Gait Studies Program, Faculty of Health Sciences, La Trobe University, Bundoora, Australia; Pure Sports Medicine, London, Australia; Centre for Sports and Exercise Medicine, Queen Mary University of London, London, UK; Department of Engineering, Aarhus University, Aarhus, Denmark; Orthopaedic Surgery Research Unit, Aalborg University Hospital, Aalborg, Denmark; Department of Occupational Therapy and Physiotherapy, Aalborg University Hospital, Aalborg, Denmark; Center for Sensory-Motor Interaction, Department of Health Science and Technology, Aalborg University, Aalborg, Denmark

**Keywords:** Navicular drop, Navicular motion, Foot kinematics, In-shoe, Stretch-sensor

## Abstract

**Background:**

Non-invasive evaluation of in-shoe foot motion has traditionally been difficult. Recently a novel ‘stretch-sensor’ was proposed as an easy and reliable method to measure dynamic foot (navicular) motion. Further validation of this method is needed to determine how different gait analysis protocols affect dynamic navicular motion.

**Methods:**

Potential differences in magnitude and peak velocity of navicular motion using the ‘stretch sensor’ between (i) barefoot and shod conditions; (ii) overground and treadmill gait; and/or (iii) running and walking were evaluated in 26 healthy participants. Comparisons were made using paired t-tests.

**Results:**

Magnitude and velocity of navicular motion was not different between barefoot and shod walking on the treadmill. Compared to walking, velocity of navicular motion during running was 59% and 210% higher over-ground (*p* < 0.0001) and on a treadmill (*p* < 0.0001) respectively, and magnitude of navicular motion was 23% higher during over-ground running compared to over-ground walking (*p* = 0.02). Compared to over-ground, magnitude of navicular motion on a treadmill was 21% and 16% greater during walking (*p* = 0.0004) and running (*p* = 0003) respectively. Additionally, maximal velocity of navicular motion during treadmill walking was 48% less than walking over-ground (*p* < 0.0001).

**Conclusion:**

The presence of footwear has minimal impact on navicular motion during walking. Differences in navicular motion between walking and running, and treadmill and over-ground gait highlight the importance of task specificity during gait analysis. Task specificity should be considered during design of future research trials and in clinical practice when measuring navicular motion.

## Background

Excessive or abnormal foot pronation mechanics are thought to result in lower extremity mal-alignment and pathology due to joint coupling with the tibia and femur [1,2]. Based on this theory, several clinical measures of foot pronation have been proposed, attempting to identify those at greatest risk of injury and to guide treatment decisions (e.g. foot orthoses prescription). The reason for multiple proposed measures of foot pronation may be due to the controversy related to which segments and planes of movement are associated. Importantly, there is also large variation in foot motion between individuals in relation to how specific joints move and interact with each other [3].

One common clinical method to measure foot pronation proposed by Brody [4], static navicular drop, involves calculation of the difference in vertical navicular height between sub-talar joint neutral and relaxed standing positions [4]. Menz [5] proposed that the addition of navicular drift or “medial bulging” should be combined with navicular drop measurement. Additionally, he proposed these measures may offer significant benefits over more traditional measures of foot pronation such as rearfoot angle, providing an indication of talonavicular (midfoot) motion [5]. Although, there is a paucity of research evaluating the link between navicular drift and lower limb injury, current evidence indicates greater navicular drop may be a risk factor for both medial tibial stress syndrome (MTSS) [6] and patellofemoral pain (PFP) [7]. However, associated effect sizes are small [6,7], possibly owing to the multifactorial nature of these conditions. Additionally, static navicular drop is a poor predictor of dynamic foot function [8], taking into account only sagittal plane motion without consideration to other planes of movement [5].

Non-invasive measurement of in-shoe foot motion has been traditionally difficult, due to the need for skin-marker placement over the foot. One alternative is to place markers over the shoe, however, previous research indicates this method may poorly reflect true foot motion. Previous studies have reported both over- [9,10] and under-estimation [11] of actual foot motion when measured by markers on the shoe. Shoe windows have also been cut, allowing marker placement, but this affects the shoes structural integrity [12], and is not feasible in a clinical setting. More recently, Christensen et al. [13] proposed the use of a ‘stretch-sensor’ as an easy and efficient method to measure and provide insight into shod and barefoot tri-planar navicular motion. This may prove to be a valuable tool in identifying injury risk, guiding treatment decisions, and measuring the effectiveness of interventions designed to control navicular motion. However, further validation is needed to determine how different gait analysis protocols affect dynamic navicular motion.

Evaluation of running and walking biomechanics is frequently completed on a treadmill. Previously, most research has focused on sagittal plane differences and has measured predominantly proximal motion. Studies comparing treadmill to over-ground gait indicate decreased peak and range of knee flexion during both walking and running on a treadmill [14-19]; inconsistent differences for hip flexion during running with both increased [20,21] and decreased [19] peaks on a treadmill; decreased ankle dorsiflexion range of motion, velocity and peak [19,22] when running on a treadmill; and greater rearfoot/ankle eversion during running (1.5 – 6.3^0^) [18,19,23] on a treadmill, although one study did report no significant difference in rearfoot motion [22]. To the author’s knowledge, there does not appear to be any comparison of midfoot or navicular motion during treadmill and over-ground walking or running. Additionally, no previous research has compared navicular motion between barefoot and shod conditions, or between walking and running.

This study investigates whether differences in magnitude and peak velocity of navicular motion exist between (i) running and walking; (ii) overground and treadmill gait; and/or (iii) barefoot and shod conditions. It was hypothesised that magnitude and velocity of navicular motion would be greater during running compared to walking.

## Methods

This cross-sectional study compared navicular motion during five different test conditions in 26 (11 female and 15 male) participants. Mean (standard deviation) for age, height, weight, and foot length of participants was 27 (7) years, 178 (9) cm, 69 (11) kg and 25.3 (1.9) cm respectively. The 26 participants (age 19-57) were conveniently sampled from the School of Engineering, Aarhus University, and surrounding community. Prospective participants with present injury or pain in the lower extremities or back preventing them from walking or running; or with medical or neurological conditions were excluded. The study was approved by Aarhus University and conducted in accordance with the Helsinki Declaration [24] and all participants were given written and verbal information about the project and signed an informed consent before participating.

### Measure of dynamic navicular motion (using the stretch-sensor)

**Figure 1:**
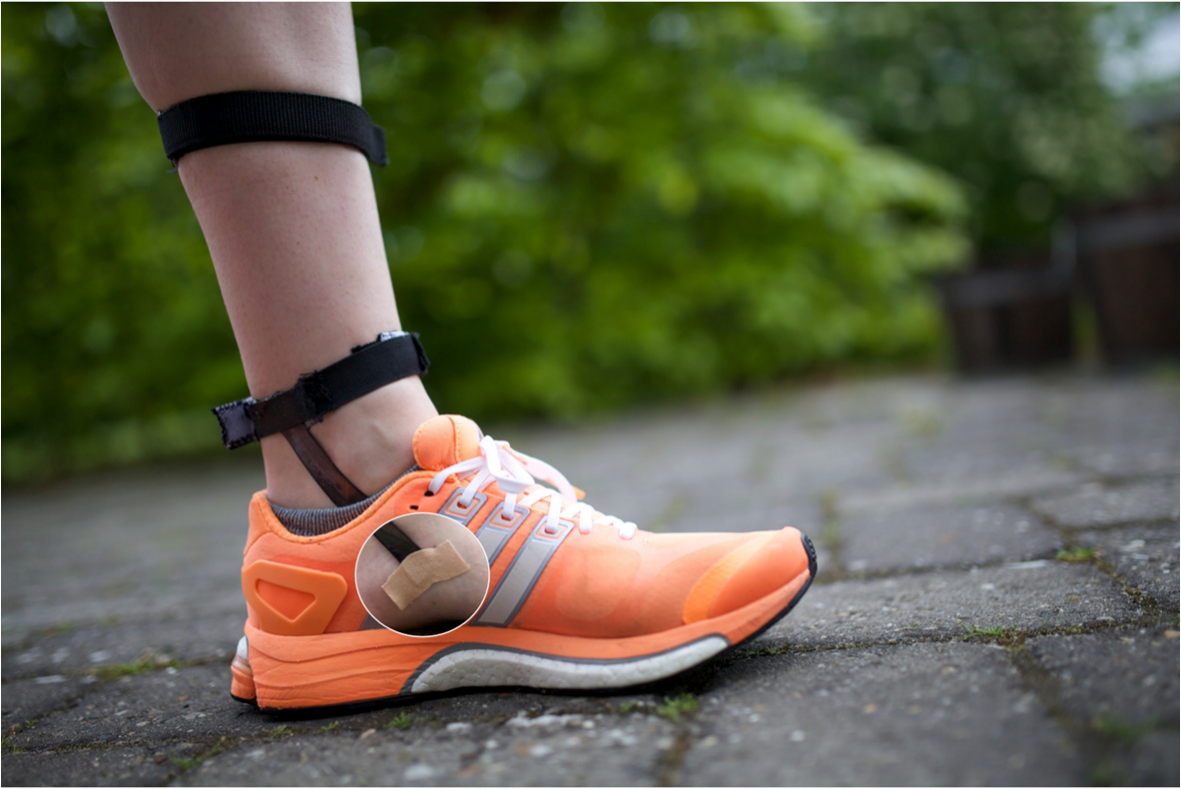
**Placement of the stretch sensor.**

The stretch-sensor is a flexible and thin capacitive sensor, allowing measurement of in-shoe navicular motion. Two points on the medial aspect of the foot are used, including 20 mm posterior to the medial malleolus (secured using a Velcro strap) and 20 mm posterior and 20 mm distal to the navicular tuberosity (Figure 1) [25]. Attachment point choices were based on a pilot study [25]. The prominence of the medial malleolus prevented positioning the distal part of the stretch-sensor directly onto the navicular bone, hence the posterior and distal location (Figure 1). Based on previous bone-pin studies by Wolf *et al.* [26] indicating the entire medial midfoot moves in the same direction during walking, it is believed this position is likely a good proxy of functional navicular drop and drift [13,27].

### Data acquisition device

The data acquisition device was custom-made hardware that enabled recording of the elongation of a stretch sensor with a sample rate of 200Hz. The device contained a three axis gyroscope and a three axis accelerometer that was sampled simultaneously with the measurements stored on a SD card together with the data from the stretch sensor. The device was mounted just above the malleoli to minimise the movement of the device caused by contractions of the muscles in the lower leg.

### Test conditions

All participants followed the same order of test conditions. Acclimatisation was determined by both visual observation by the investigator and participants answering ‘yes’ when asked “do you feel that you are walking or running as you normally would?” Test conditions in order were as follows:

### Participant’s own standard athletic trainers and socks

1. Self- selected over-ground walking pace
2. Self-selected over-ground running pace
3. Treadmill walking (same pace as during over-ground walking)
4. Treadmill running (same pace as during over-ground running).

### Barefoot (with socks)

5. Treadmill walking (same pace as during over-ground walking).

### Outcome measurement

Primary outcomes included the magnitude and peak velocity of navicular motion measured by the stretch sensor. Velocity of navicular motion (i.e. peak velocity) was estimated by differentiating the elongation of the stretch-sensor, specifically convoluting the elongation of the stretch-sensor with the filter mask [-0.5 0 0.5]. The intra and intertester reliability of the sensor has previously been reported as acceptable with ICC(2.1) > 0.76 for barefoot measurements and ICC(2.1) 0.65 for shod measurements [13]. Additionally, conservative estimates for 95% limits of agreement for intra- and inter-rater reliability have been established as between -2.4 and 2.6 mm [13]. Testing was completed by the same tester, who had used the stretch-sensor approximately 100 times prior to study commencement.

### Sample size

The sample size was based on pilot data and aimed at detecting a 20% difference in navicular motion between shod overground walking and running. Using a standard deviation of 30%, power of 80% and alpha at 0.05, at least 20 participants were needed.

### Data analysis

**Figure 2:**
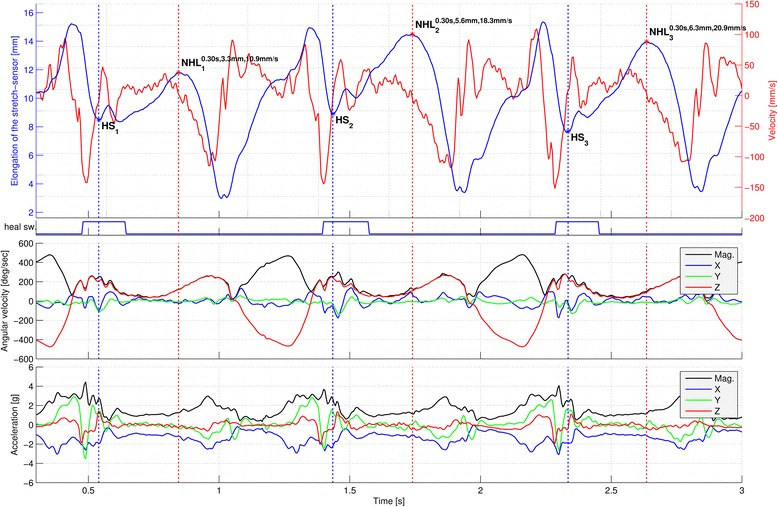
**Raw data of magnitude (measured as difference between HS and NHL) and velocity of navicular motion measured by the stretch sensor from one participant over three steps.** HS = heel strike; NHL = navicular height loaded.

**Figure 3:**
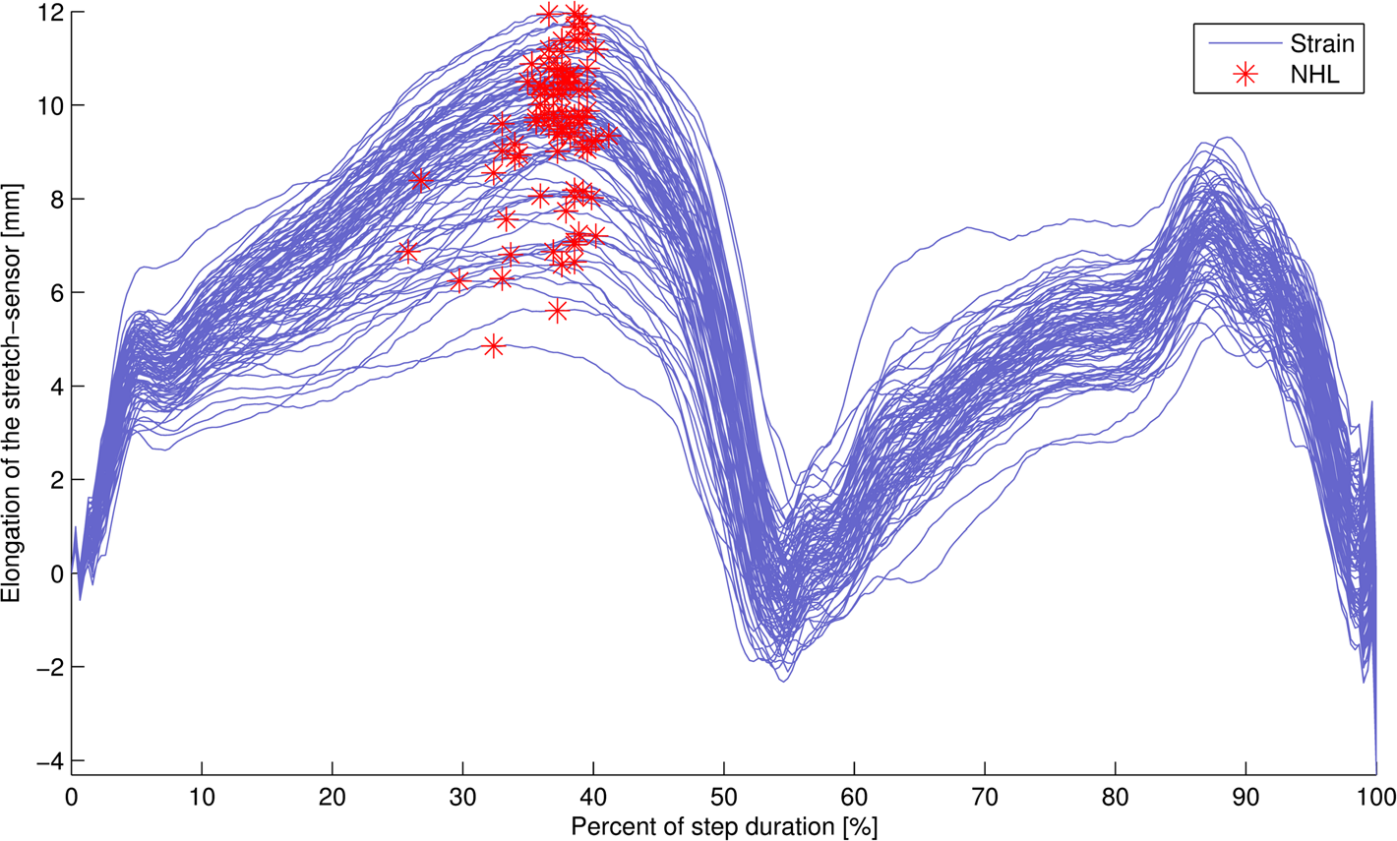
**Variation in navicular motion across different stance phases for one participant.**

Data were analysed using a custom-written Matlab script. Heel strike for each stance phase was manually determined using data from the accelerometer and gyroscope, which has excellent reliability and validity [28]. As security, this data was checked against the data from a pressure sensitive heel switch. Afterwards, a custom written algorithm determined the maximal magnitude of navicular motion for each stance phase. An example of raw data is illustrated in Figure 2. Magnitude of navicular motion was calculated as the difference between elongation of the stretch-sensor at heel-strike and maximal elongation during stance. The average of the maximal estimated velocity in the stance phase over all steps was calculated. Due to considerable step to step variation (Figure 3), the average magnitude of navicular motion during 53-156 consecutive steps depending on condition and participant cadence was calculated.

### Statistical analysis

Separate one way analysis of variance (ANOVA) were completed for magnitude and velocity of navicular motion, with paired samples t-tests used to compare differences between test conditions. Bonferroni adjustment was not made for pairwise comparisons to ensure potentially clinically meaningful findings were not missed due to stringent statistical correction [29]. Effect size (ES) were calculated using the mean difference between conditons divided by the standard deviation of the first condition [30]. Additionally the percentage of differences were calculated for each comparison made. Following criteria proposed by Hume *et al*. [31], ES were allocated as small (<0.6), medium (0.61 – 1.19), or large (≥1.2). Pearson’s product moment correlation evaluated the association between the magnitude and velocity of navicular motion between test conditions.

## Results

Data of one participant was excluded from all trials due to poor data quality. Additionally, another participant was excluded from treadmill running, and four other participants were excluded from barefoot treadmill walking due to poor data quality. ANOVA results indicated a significant effect of condition on magnitude of navicular motion (F = 6.9, *p* = 0.002) and on velocity of navicular motion (F = 52.6, *p* = <0.001).

### Barefoot versus shod treadmill walking

Magnitude and velocity of navicular motion was similar between barefoot and shod walking on the treadmill (Table 1). There was a significant association between magnitude and velocity of navicular motion during shod and barefoot walking on a treadmill (r = 0.80, *p* < 0.0001), (r = 0.61, *p* = 0.003) and respectively.

**Table 1:** **Navicular motion and velocity of navicular motion during over ground walking and running and during treadmill walking and running**

	**Treadmill**			**Over-ground**		**TrW Vs TrBF**	**TrR Vs TrW**	**OGR Vs OGW**	**TrW Vs OGW**	**TrR Vs OGR**
	**TrW**	**TrR**	**TrBF**	**OGW**	**OGR**	**Difference (95% CI)**	**Difference (95% CI)**	**Difference (95% CI)**	**Difference (95% CI)**	**Difference (95% CI)**
	**(n = 25, 72-134 steps)**	**(n = 24, 108-156 steps)**	**(n = 21, 80-156 steps)**	**(n = 25, 53-72 steps)**	**(n = 25, 64-117 steps)**					
Navicular Motion – mm (95%CI)	8.5	9.9	8.0	7.0	8.6	0.5^	1.2^	1.6^§^	1.5^	1.4^
	(7.0;10.1)	(8.3;11.5)	(6.6;9.4)	(5.7;8.3)	(7.6;9.6)	(-0.5 to 1.5)	(-0.1 to 2.4)	(0.3 to 2.9)	(0.8 to 2.3)	(0.5 to 2.3)
						p = 0.34	p = 0.07	p = 0.02	p = 0.0004	p = 0.003
Velocity* - mm/s (95%CI)	130	394	142	249	396	18^	273^#^	148^§^	119^#^	2^
	(97;164)	(322;465)	(118;167)	(207;292)	(337;455)	(-8 to 44)	(216 to 330)	(117 to 179)	(86 to 152)	(-22 to 26)
						p = 0.16	p < 0.0001	p < 0.0001	p < 0.0001	p = 0.85

*Mean Peak velocity.

^#^ = large effect size (≥1.20); ^§^ = medium effect size (0.60 – 1.19); ^ = small effect size (<0.60).

TrW = treadmill walking; TrR = treadmill running; TrBF = treadmill barefoot walking; OGW = over-ground walking; OGR = over-ground running; 95% CI = 95% confidence interval).

### Running versus walking

Compared to walking, velocity of navicular motion during running was 59% (ES = 1.03) and 210% (ES = 1.63) higher over-ground and on a treadmill respectively (see Table 1). Magnitude of navicular motion was 23% (ES = 0.64) higher during over-ground running compared to over-ground walking. Additionally, there was a statistical trend (*p* = 0.07) towards a 14% (ES = 0.31) increase in the magnitude of navicular motion during treadmill running compared to treadmill walking.

### Over-ground versus treadmill walking and running

Compared to over-ground, magnitude of navicular motion on a treadmill was 21% (ES = 0.39) and 16% (ES = 0.36) greater during walking and running respectively (see Table 1). Compared to over-ground, maximal velocity of navicular motion was 48% (ES = 1.45) less during walking on a treadmill, but there were no differences in maximal velocity during running between the two conditions (see Table 1).

There were significant associations between magnitude (r = 0.88, *p* < 0.0001) and velocity of navicular motion (r = 0.66, *p* = 0.003) during over-ground and treadmill walking. Magnitude (r = 0.88, *p* < 0.0001) and velocity (r = 0.95, *p* < 0.0001) of navicular motion during over-ground running was strongly associated with treadmill running.

## Discussion

Traditionally, the need to place markers directly over the foot has made measuring in-shoe foot motion difficult. In this study, a viable method of measuring in-shoe navicular motion was used to identify possible differences between barefoot and shod walking, running and walking, and between treadmill and over-ground gait. Overall, these findings highlight the need for task specificity during gait analysis of navicular motion, with differences between running and walking, and treadmill and over-ground gait indicated.

Magnitude and velocity of navicular motion is greater during running compared to walking, regardless of whether comparison is made on a treadmill or over-ground. Specifically, small to moderate increases in magnitude, and moderate to large increases in velocity were found, indicating running is likely to have a greater effect on velocity than magnitude of navicular motion. Greater magnitude and velocity of navicular motion is likely the result of greater vertical ground reaction forces occurring during running [32,33]. Importantly, greater navicular motion during running highlights the need for task specificity when evaluating foot function in a research or clinical setting.

Evaluation of running and walking biomechanics is frequently completed on a treadmill. Previous research has indicated likely differences between treadmill and over-ground gait at the hip [19-21], knee [14-19], and ankle/rearfoot [19,22]. To the author’s knowledge, this is the first study to compare midfoot motion between treadmill and over-ground walking or running. Although a large reduction of navicular motion velocity occurred during treadmill walking, moderate to large increases in magnitude of navicular motion resulted during treadmill walking and running compared to over-ground. Importantly, similar to sagittal plane motion at the hip, knee and ankle, this indicates navicular motion on a treadmill may not accurately reflect over-ground conditions, particularly during walking. This should be considered during gait assessment in both research and clinical practice settings.

Greater navicular motion during treadmill gait in this study is consistent with increases in ankle eversion (1.5 – 6.3^0^) previously reported during treadmill running [18,19,23]. This consistency between different studies is logical, since navicular and rearfoot motion may both be considered proxies for foot pronation [5], and strong correlations between the two have been previously reported [34]. Reduced velocity of navicular motion during walking on a treadmill compared to over-ground is also consistent with previous reports of reduced internal inversion moments during treadmill walking [14], which is a measure of forces required to control foot pronation. Further research concurrently collecting stretch sensor data with ground reaction forces, muscle function and more proximal kinematics is needed to understand potential mechanisms for the differences in navicular motion between treadmill and overground gait.

Foot motion during gait is commonly measured barefoot due to issues with marker placement on the shoe [12]. This method is often criticised as barefoot may not accurately reflect shod kinematics. Findings in this study indicate the magnitude and velocity of navicular motion was not significantly different between barefoot and shod treadmill walking. Additionally, there were strong correlations between the two conditions for both magnitude and velocity of navicular motion, indicating footwear may have minimal impact on navicular motion during walking. Whilst walking findings from this study align with previous bone-pin research indicating minimal difference in calcaneal and tibial movement patterns between barefoot and shod running [35], other research indicates barefoot running may be associated with reduced and earlier rearfoot eversion [36]. Additionally, increased step rate, reduced ankle dorsiflexion, and lower vertical ground reaction forces have also been reported during barefoot running [37]. Further research is needed to explore if differences in navicular motion exist between barefoot and shod running, and the potential implications of this.

### Limitations and future research

Poor data quality for some conditions led to the exclusion of some participant data. Specifically, data for treadmill running was excluded in one participant, and data from four other participants were excluded from barefoot treadmill walking. Poor data quality in these instances is attributed to accumulation of sweat during testing, and subsequent loosening of the stretch sensor attachments, with the data lost coming from the final two conditions tested. Poor data quality was determined by very large navicular drop, e.g. 20 or 30mm, a velocity of more than 2000 mm/s, or a loss of the normal rhythm (see Figure 2). To address this in future research, the use of skin adhesive, evaluating fewer conditions, and/or reapplying and recalibrating the ‘stretch-sensor’ during testing is recommended.

Participants performed testing conditions in a set order to allow matching of outdoor walking and running speed. This may have led to systematic differences between conditions as result of fatigue [38], potentially limiting generalisability of findings. However, Dierks et al. [39] has previously reported greater excursion and peak velocity of rearfoot eversion during running following an exhaustive running protocol, which averaged 45 minutes and all 20 runners reached a rated perceived exertion of 15. Increases were 1.2^0^ (15%) and 12.8^0^ per second (11%) respectively. Considering the protocol used in the current study was far less fatiguing, and differences identified were of larger magnitudes (16 – 210%), it is unlikely that fatigue substantially contributed to current findings. Additionally, unpublished data indicates that navicular motion measured by the stretch sensor remains consistent until at least 30 minutes of fast barefoot walking. Nonetheless, further research is warranted to confirm findings from this study, and to understand the impact of fatigue on navicular motion measured by the stretch sensor during various conditions. Aclimatisation to treadmill running varied between two and five minutes, and not six minutes recommended by Matsas [17]. However, all participants reported feeling comfortable in each testing condition prior to commencement of data collection. Further research is needed to determine an adequate aclimitisation period to ensure consistent navicular motion during treadmill gait.

Due to the anatomical placement of the stretch sensor over the navicular, it is thought to provide insight into tri-planar talonavicular (midfoot) motion during gait [5]. However, further research comparing motion measured using the stretch sensor to three dimensional motion of the midfoot and its individual components including the talonavicular joint, as well as rearfoot and forefoot segments is needed to understand the possible biomechanical information it can provide.

## Conclusion

Measurement of navicular motion using the ‘stretch-sensor’ is a reliable method of measuring in-shoe navicular motion during gait, and can detect differences between varied gait analysis protocols. Magnitude and velocity of navicular motion is higher during running compared to walking, magnitude of navicular motion is higher during walking and running on a treadmill compared to over-ground, and velocity of navicular motion is lower during walking on a treadmill compared to overground. These differences highlight the importance of task specificity during gait analysis, and should be considered during design of future research trials and during gait assessment in clinical practice.
